# Basal ganglia neurons in healthy and parkinsonian primates generate recurring sequences of spikes

**DOI:** 10.1152/jn.00265.2022

**Published:** 2023-04-05

**Authors:** Adriana Galvan, Thomas Wichmann

**Affiliations:** ^1^Emory National Primate Research Center, Emory University, Atlanta, Georgia, United States; ^2^Udall Center of Excellence in Parkinson’s Disease Research, Department of Neurology/School of Medicine, Emory University, Atlanta, Georgia, United States; ^3^Aligning Science Across Parkinson’s (ASAP) Collaborative Research Network, Chevy Chase, Maryland, United States

**Keywords:** basal ganglia, globus pallidus, parkinsonism, primate, subthalamic nucleus

## Abstract

The spiking activity of basal ganglia neurons can be characterized by summary statistics such as the average firing rate, or by measures of firing patterns, such as burst discharges, or oscillatory fluctuations of firing rates. Many of these features are altered by the presence of parkinsonism. This study examined another distinct attribute of firing activity, i.e., the occurrence of repeating sequences of interspike intervals (ISIs). We studied this feature in extracellular electrophysiological recordings that were made in the basal ganglia of rhesus monkeys, before and after they had been rendered parkinsonian by treatment with the neurotoxin 1-methyl-4-phenyl-1,2,3,6-tetrahydropyridine. Neurons in both pallidal segments and in the subthalamic nucleus tended to fire in repeating sequences, typically two ISIs long (i.e., involving three spikes). In recordings that were 5,000 interspike intervals long, 20%–40% of spikes participated in one of many sequences with each ISI replicating the sequence pattern with a timing error of ≤1%. Compared with similar analyses in shuffled representations of the same data, sequences were more common in the original representation of ISIs in all of the tested structures. Induction of parkinsonism reduced the proportion of sequence spikes in the external pallidum but increased it in the subthalamic nucleus. We found no relation between the sequence generation and the firing rate of neurons, and, at most, a weak correlation between sequence generation and the incidence of bursts. We conclude that basal ganglia neurons fire in recognizable sequences of ISIs, whose incidence is influenced by the induction of parkinsonism.

**NEW & NOTEWORTHY** Previous work has shown that the timing of the electrical activity of basal ganglia neurons has nonstochastic properties, resulting in oscillatory firing patterns, or bursting. This article describes another such property in the monkey brain; a surprisingly large proportion of action potentials generated by cells in the extrastriatal basal ganglia are part of precisely timed recurring sequences of spiking events. We also found that the generation of these sequences changes substantially in the parkinsonian state.

## Graphical abstract

**Figure:**
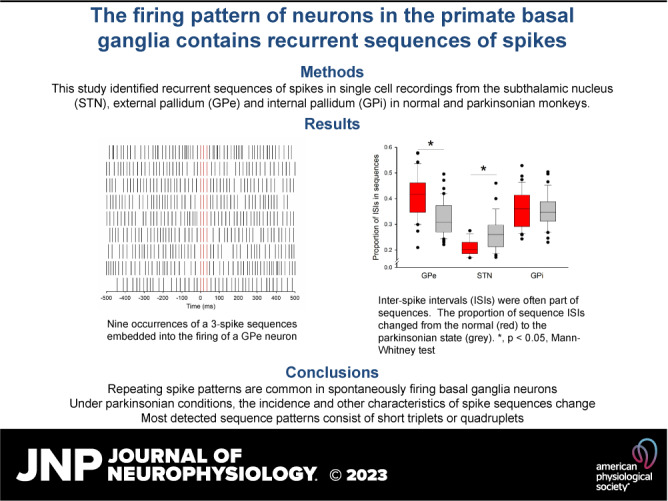

## INTRODUCTION

Early electrophysiological studies of the spiking activities of spontaneously active basal ganglia neurons in primates have focused on summary statistics such as the average firing rate, its variability, or the distribution of interspike intervals (ISIs). These statistical measures assume that the occurrence of individual action potentials is independent of others, i.e., that each action potential resets a stochastic process according to which the next action potential is triggered. More recent studies of basal ganglia activity have instead focused on firing behavior that is not governed by a random process, and leads to recognizable firing patterns, such as oscillatory fluctuations of firing rates, the presence of clusters of short ISIs (“bursts”), or fractality (1–7). Changes in both, the summary parameters and the parameters describing firing patterns, have been identified in animal models of basal ganglia diseases, such as the dopamine-depletion models of primate parkinsonism produced by injections of the neurotoxin 1-methyl-4-phenyl-1,2,3,6-tetrahydropyridine (MPTP) (summarized in Ref. 3). In such animals, neurons in the subthalamic nucleus (STN), the external and internal pallidal segments (GPe and GPi, respectively), and the substantia nigra pars reticulata (SNr) show a rise in the incidence of burst discharges (3, 8–11), and the emergence of prominent low-frequency oscillatory firing patterns, synchronized across neighboring cells (12, 13). Some of these findings have also been identified in human patients with Parkinson’s disease who underwent brain recording sessions as part of functional neurosurgical procedures (14–17).

Identifying features of firing patterns such as bursts or oscillations in individual neurons implies that the temporal order of ISIs of these neurons is governed by cellular or network dynamics that favor the occurrence of statistically improbable arrangements of ISIs. In the current work, we examine this issue further, by studying whether neurons in the primate basal ganglia show (statistically improbable) exactly repeating patterns of ISIs, and whether the prevalence of such repeated sequences is influenced by dopamine depletion. Similar repeating patterns of discharge were previously identified in sensory systems (18–24), often occurring in relation to, or representing, the occurrence of specific sensory stimuli. Our study of data from normal and parkinsonian primates shows that strictly repeating sequences of ISIs are also a feature of spontaneous firing in the normal/healthy basal ganglia and that parkinsonism alters the prevalence and duration of such sequences.

## METHODS

### General Study Design

**Figure 1.**
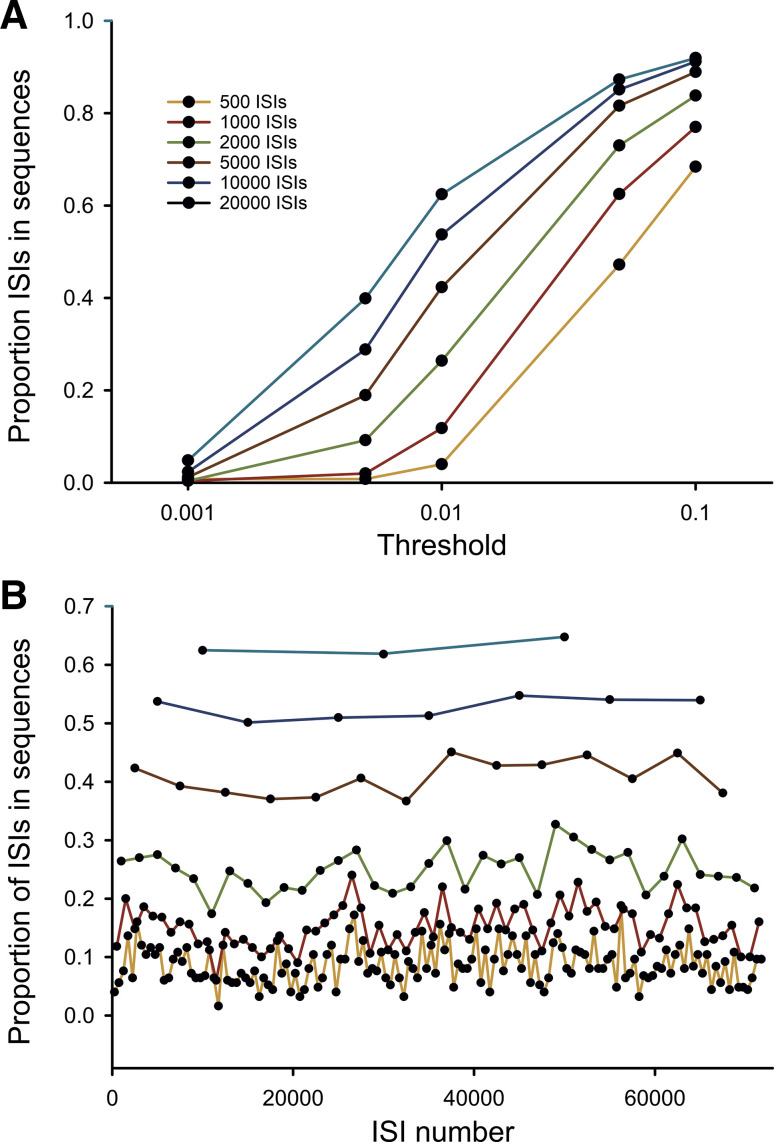
Method of sequence detection. *A*: influence of threshold and length of record on the overall proportion of spikes detected to be contained in sequences as defined in methods. The abscissa shows the “threshold” parameter [variability in interspike interval (ISI) duration (respective to the ISI length) for an ISI to be accepted as being part of a sequence], and the ordinate the corresponding proportion of spikes identified to be in sequences. The different curves were generated with differing lengths of ISI series (color code indicated in figure), using data from from a single example cell in the external pallidal segment (GPe). *B*: stability of results of sequence detection. The data were generated using data from a GPe cell that contained more than 70,000 spikes. Data were broken into segments of different lengths, and separately analyzed to yield the proportion of ISIs in sequences (ordinate). Same color code as in *A*.

We conducted electrophysiological recordings of the spontaneous single-cell activities in the GPe, GPi, and STN in rhesus macaques, before and after systemic treatment of the animals with MPTP, inducing a permanent parkinsonian state. The source data are a subset of previously published data (5, 11), including all records that contain at least 5,000 ISIs. The first 5,000 ISIs of such records were analyzed to study the occurrence of repeating patterns of ISIs. Data from individual cells for which longer records were available were also analyzed, to study some of the characteristics of the sequence detection process (as shown in Fig. 1).

### Animals

The data are based on electrophysiological recordings that were carried out in two rhesus monkeys (*Macaca mulatta*, 4–5 kg). The animals were housed under conditions of protected contact housing, with free access to standard primate chow, water, and supplemental fruit and vegetables. Prior to the recording sessions, the animals were trained to adapt to the laboratory environment and to sit in a primate chair and permit handling by the experimenter. All experiments were performed in accordance with the NIH Guide for the Care and Use of Laboratory Animals, the Public Health Service Policy on the Humane Care and Use of Laboratory Animals, and the American Physiological Society’s Guiding Principles in the Care and Use of Animals. All experiments were approved by the Institutional Animal Care and Use Committee of Emory University.

### Surgical Procedures

After initial behavioral conditioning, the animals underwent a surgical procedure under aseptic conditions and isoflurane anesthesia (1%–3%). Standard stainless steel recording chambers were stereotactically positioned over trephine holes. The animals then received two chambers, one directed at the pallidum (GPe, GPi), placed at a 50° angle from the vertical in the coronal plane, and one directed at the STN, using a 36° angle from the vertical in the sagittal plane. These chambers were affixed to the skull with dental acrylic, along with stainless steel head holders.

### Administration of MPTP and Assessment of Parkinsonism

After completion of recordings in the normal state, the animals received MPTP. The toxin was injected under general isoflurane anesthesia (1%–3%) into the right common carotid artery with the external carotid artery occluded, so that the toxin reached the brain via the internal carotid artery (0.5 mg/kg per injection; one monkey received 2 injections separated by 2 wk, while the other received a single injection). Both animals developed similarly obvious signs of moderate parkinsonism (bradykinesia, rigidity, and flexed posturing of arm and leg) contralateral to the injections. The animals did not receive dopaminergic medications. The recordings in the parkinsonian state started 2 mo after the MPTP treatment. Throughout the post-MPTP period, the behavioral state of the animals remained stable, as assessed with routine behavioral observations (11, 25, 26). After stable parkinsonism was established, electrophysiological recordings resumed on the animal’s right side (ipsilateral to the MPTP administration).

### Histology

At the conclusion of the experiments, the monkeys were euthanized by induction of deep anesthesia with an overdose of pentobarbital sodium, followed by transcardial perfusion with saline and 4% paraformaldehyde in 0.1 M phosphate buffer (pH 7.2). The brains were removed and cryoprotected in 30% sucrose solution in 0.1 M phosphate buffer. The fixed brains were sectioned in coronal planes (50 µm). One of every four sections was stained with cresyl violet for localization of microelectrode tracks. The results of the histological examinations are documented in our previous publication (11), showing the placement of the recording electrodes in the basal ganglia, minimal overall damage from the electrode penetrations, as well as the MPTP-treatment-induced striatal dopaminergic denervation, based on immunohistochemistry.

### Electrophysiological Recordings

The electrophysiological recordings were done with the animals seated in a standard primate chair, with their head restrained. Recordings were conducted during episodes when the animals were fully awake, as verified by direct observation. The neuronal activity of basal ganglia neurons was recorded with commercial tungsten microelectrodes (Frederick Haer Co., Bowdoinham, ME; impedance 0.5–1.0 MΩ at 1 kHz). The electrodes were lowered into the brain with a microdrive (MO-95B, Narishige, Tokyo, Japan), using a guide tube. The electrical signals were amplified (DAM-80 amplifier, WPI, Sarasota, FL), filtered (400–10,000 Hz, Krohn-Hite, Brockton, MA), displayed on a digital oscilloscope (DL1540, Yokogawa, Tokyo, Japan), made audible via an audio amplifier, and recorded as digital signals. Neurons in the GPe, GPi, and STN were identified based on their stereotaxic location within the brain, the regional relationship to nearby nuclei, and their characteristic firing patterns, including the findings of neuronal firing at high-frequency with pauses in the GPe, the finding of high-frequency discharging cells without pauses in the internal pallidal segment, and the finding of cells firing at lower rates embedded into an area of high background activity in the STN (see, e.g., Refs. 27 and 28). The location of neurons was subsequently reconstructed based on stereotaxic information, micromanipulator readings during the recordings, and the results of postmortem histological analysis. For initial inclusion into the analysis, cells had to be adequately isolated throughout the record, as defined by a signal-to-noise ratio of at least 3. The recorded activity was later replayed and processed with a template-matching spike-sorting device (Alpha-Omega, Nazareth, Israel), which extracted the timing of spike occurrence. The data were then stored as series of ISIs.

### Data Analysis

The ISI data were imported into the Matlab computing environment (Mathworks, Natick, MA) for further analysis. Using a custom-written Matlab algorithm, we analyzed whether repeating sequences of two or more ISIs could be found in the data stream. A sequence of ISIs was said to repeat itself if the duration of each member of the repeated sequence was within 1% of the corresponding value within the original sequence. In supplementary analyses, we also examined the use of other cutoff values, including 5%, 0.5%, and proportional cutoff values adjusted using the median ISI of the respective cell, to represent a maximal error of either 1, 2, or 4 ms.

In a subsequent step of the algorithm, we examined whether the identified sequences were overlapping or fully contained in one another. Sequences that were fully contained in longer ones were removed. If some repetitions of a short sequence were found to be contained in a larger one, while other repetitions occurred in isolation, the isolated occurrences were retained if they were found more than once. If some instances of the detected sequences overlapped with others, the longer overlapping sequence was retained in the record, while the remaining member(s) of the shorter overlapping sequence(s) were discarded.

We considered that any (random) arrangement of ISIs from given distributions may lead to a finding of repeating sequences. To determine the probability of finding repeating sequences as a random process, given a neuron’s ISI distribution, we applied the steps of identifying sequences (as described earlier) to 1,000 randomly arranged representations of the same ISI data for each cell.

The data based on the original ISI stream and those generated from each of the shuffled representations of a cell’s data were used to calculate the total number of sequences, the distribution of sequence length, the proportion of spikes and time spent in sequences, and the median duration of sequences. The results based on the original data were also expressed in proportion to the results from the shuffled data.

Focusing on 2-ISI sequences (the most frequent type of sequences), we also measured the maximal number of repetitions of a given sequence in each cell. To analyze whether a given cell’s sequences were evenly distributed across the data stream, or whether they were systematically clustered, we calculated the median intersequence distance (number of ISIs between recurrences of the sequences, normalized to the expected distance length, based on the number of recurrences of the sequence) and compared the results with those generated in shuffled data. To study whether the detected sequences have a recognizable stereotypic temporal structure, we divided the duration of ISIs occurring within 2-ISI sequences by the cell’s median ISI duration. Ratios between the second and first ISI of such sequences were also analyzed.

Given the possibility that the finding of bursts may overlap with the finding of sequences, we were also interested in examining how the incidence of sequences corresponded to the occurrence of bursts. We identified the presence of bursts with the “surprise” algorithm (29), using a cutoff-surprise value of 3, allowing us to identify those ISIs that were part of bursts. We then determined the proportion of sequence ISIs that were also identified as burst ISIs and the proportion of burst ISIs that were also found within sequences. Regression analyses were performed to investigate correlations between the incidence of bursts and that of sequences across the population of neurons.

### Statistics

We used the paired nonparametric Wilcoxon test to compare the original proportion of spikes in sequences and the proportion of time spent in sequences with the median of the results based on shuffled data of each cell. These analyses were done separately for the pre- and post-MPTP data. Comparisons between the pre- and post-MPTP data relied on unpaired nonparametric Mann–Whitney tests. For all tests, a criterion of *P* < 0.05 was taken as a measure of significance. All numerical data are shown as medians with interquartile ranges.

## RESULTS

### Database

The number of cells presented in this report and the average duration of the records are shown in Table 1. Sufficient data from a total of 78 neurons from GPe (38 recorded in the normal state, 40 recorded in the MPTP-treated state), 67 neurons from GPi (33 recorded in the normal state, 34 recorded in the parkinsonian state), and 40 neurons from STN (13 recorded in the normal state, 27 recorded in the parkinsonian state) were available. Table 1 also provides information about the firing rate and the coefficient of variation (CV) of the ISIs. We found that the cells recorded in parkinsonian state in the GPe were significantly slower than those recorded in the normal state. The neurons in the STN fired significantly faster. The firing rate in GPi in the MPTP-treated state was higher than that in normal state, but this difference was not significant. The average coefficient of variation (CV) of ISI durations did not differ between the two states in any of the three regions.

**Table 1.** Basic firing characteristics of neurons in GPe, STN, and GPi

Structure	State	Number of ISIs	Duration, s	Firing rate, spikes/s	CV of ISIs
GPe	Normal (*n* = 38)	31,967 (17,629)	530.7 (299.1)	66.6 (24.7)	1.13 (0.63)
	MPTP (*n* = 40)	19,322 (13,723) (*P* = 0.002)	474.9 (278.5) (*P* = 0.353)	45.7 (27.7) (*P* = 0.000)	1.19 (0.52) (*P* = 0.187)
STN	Normal (*n* = 13)	9,731 (7,033)	529.2 (193.6)	22.9 (10.9)	1.46 (0.42)
	MPTP (*n* = 27)	20,554 (12,452) (*P* = 0.000)	598.6 (162.7) (*P* = 0.628)	36.8 (15.8) (*P* = 0.000)	1.32 (0.41) (*P* = 0.391)
GPi	Normal (*n* = 33)	38,918 (20,919)	577.3 (194.3)	74.4 (24.7)	0.91 (0.27)
	MPTP (*n* = 34)	38,306.5 (22,959) (*P* = 0.821)	563.2 (235.7) (*P* = 0.334)	75.3 (17.7) (*P* = 0.444)	1.0 (0.49) (*P* = 0.156)

*n* values refer to the number of neurons available for study in each category*. P* values are based on Mann–Whitney tests, comparing the respective data from the normal and parkinsonian states. Data are shown as medians (IQR). CV, coefficient of variation; GPe, external pallidal segments; GPi, internal pallidal segments; ISI, interspike interval; MPTP, 1-methyl-4-phenyl-1,2,3,6-tetrahydropyridine; STN, subthalamic nucleus.

### Results of Sequence Analysis

The detection of sequences of ISIs in the recorded material was strongly influenced by the threshold chosen for detection of such sequences, as demonstrated in Fig. 1*A*, which shows the example of a single GPe neuron recorded in the normal state for which a long (72,182 spikes, recorded over 842.3 s) record was available. This GPe neuron was selected for the example because it had the longest ISI sequence available from the cells in our database, which allowed sequence detection across a broad range of data segment lengths. It can be seen that a sequence acceptance threshold of 0.001 (i.e., acceptance of ISIs to be in sequences only if their ISI durations differed by less than 1/1,000 of the respective ISI lengths) resulted in very low levels of detection, quantified as overall proportion of ISIs in sequences, among all recorded ISIs, whereas use of a threshold level of 0.1 resulted in a very high proportion of ISIs in sequences. For the main part of this study, we settled on an intermediate threshold level, 0.01 (i.e., ISIs were accepted as belonging to the same sequence, if their duration differed by less than 1/100 of the corresponding ISI length in the original sequence). We provide supplementary data that analyze the outcome of the study using other cut-off criteria (see Supplemental Figs. S1 to S5).

Figure 1*A* also demonstrates that the length of the record mattered. As expected, analysis of long data segments resulted in the identification of more sequences (and thus, a greater proportion of spikes in sequences) than analysis of short data segments, likely because repeating ISI sequences that are separated in time can be detected in long records, while they may elude detection in short records. As noted earlier, we analyzed records with at least 5,000 ISIs. If more than 5,000 ISI spikes had originally been recorded, we used the initial 5,000 ISIs for the analysis.

The data in Fig. 1*B* (same color scheme as Fig. 1*A*) demonstrate the stability of the sequence detection across time. This analysis was based on the same record as Fig. 1*A*. The record was repeatedly analyzed, breaking it into nonoverlapping consecutive segments of equal lengths, ranging from 500 and 20,000 ISIs, calculating the (measured) proportion of spikes in sequences for each. There was considerable variability/uncertainty when measurements involved short sequence lengths (500–2,000 ISIs), whereas longer data segment lengths (5,000–20,000) yielded more stable results.

**Figure 2.**
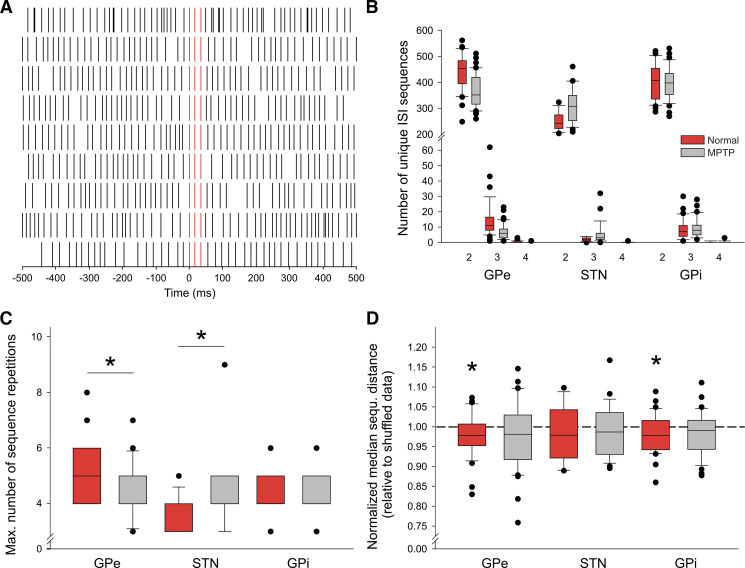
Sequence detection in the basal ganglia of normal and parkinsonian monkeys. *A*: example of repeated sequences of 2-interspike intervals (ISIs) (3 spikes) in an external pallidal segment (GPe) neurons, recorded in a normal animal. Each of the short vertical lines corresponds to a single action potential. The identified sequences were aligned to the first spike in the sequence (sequence indicated in red). *B*: overall distribution of unique sequences comprised 2, 3, or 4 ISIs in the normal (red) and parkinsonian (gray) state in GPe, subthalamic nucleus (STN), or internal pallidal segment (GPi). An analysis of the median maximal number of recurrences of 2-ISI sequences is shown in *C*. *D*: ratios between the normalized median spacing of 2-ISI sequences found in the original data, and those found in shuffled representations of the same data. Median ratios below 1 indicate that sequences are (on average) more closely spaced than explained by chance. Each cell contributed one data point. The boxplots in *B*–*D* show 25th to 75th percentile boxes, 10th and 90th percentile line indicators, and dots indicating values outside of the 10th/90th percentile boundaries. The analysis was based on the first 5,000 ISIs in each data file. *P < 0.05; Mann–Whitney test. *N* values for panels *B*, *C*, and *D* are stated in Table 1.

Figure 2*A* demonstrates the results of detection of a specific three-spike sequence of spikes in a GPe cell recorded in the normal state, different from the cell shown Fig. 1. The sequence was found nine times in this neuron’s 5,000 ISI data (original data and 8 repetitions). Such three-spike sequences (involving 2 ISIs) were, by far, the most common type of sequence identified. Figure 2*B* shows the number of unique sequences identified across cells in GPe, STN, and GPi. In this and all other summary figures, red symbols represent data from neurons recorded in the normal state, whereas gray symbols represent data from neurons recorded in the parkinsonian state. As can be seen in Fig. 2*B*, few cells showed four-spike (3-ISI) or five-spike (4-ISI) sequences. Figure 2*C* shows that the maximal repetition rates of 2-ISI sequences differed between the basal ganglia structures, with the most seen in the GPe, and the fewest in the STN. Treatment with MPTP lowered the sequence repetition in GPe and raised it significantly in the STN, while not leading to any change in the GPi. The question whether the detected 2-ISI sequences occurred in clusters was analyzed by studying the median length of intersequence distances (number of ISIs between sequences, normalized to the expected length of the distance, given its number of repetitions). The median intersequence distance would be smaller in cells with (many) tightly grouped sequences, compared with the results of shuffled representations of the data. Analysis of the ratio of the results from original and shuffled data showed that the median ratios were below 1 for all structures and states (Fig. 2*D*). This reached significance only in the sample of cells from GPe and GPi recorded in the normal state, but nevertheless suggests that sequences in the original data tended to be closer together than expected by chance (thus, formed clusters). There were no differences between the data from the normal and parkinsonian states (not shown).

### Proportion of ISIs Participating in Sequences

**Figure 3.**
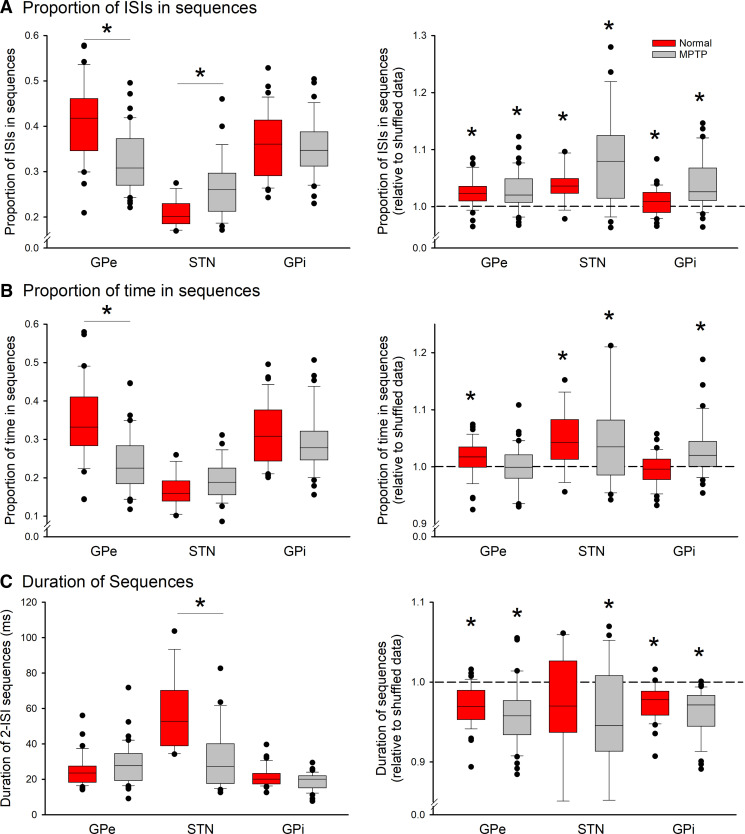
Proportion of interspike intervals (ISIs) in sequences (*A*), proportion of time spent in sequences (*B*), and duration of 2-ISI sequences (*C*). The box plots on the *left* show the median of the respective absolute values. The graphs on the *right* show the distribution of the ratios between the original data and the respective median of 1,000 shuffled renditions of the same ISI data. Cells in the external pallidal segment (GPe), subthalamic nucleus (STN), and internal pallidal segment (GPi) were recorded in the normal state (red) and in the parkinsonian state (gray). The boxes show the 25th and 75th percentile ranges, with the median marked inside of the box, the error bars show the 10th and 90th percentile, and the black dots indicating values outside of the 10th/90th percentile boundaries. Sequences were defined as described in the text. The analysis is based on the first 5,000 ISIs in each data file. *P < 0.05; Mann–Whitney test was used for plots on the *left*, comparing the data from the normal and parkinsonian states. The paired-Wilcoxon test was used for plots on the *right*, comparing the median duration of sequences of the actual data with that of the median of sequence analysis of the shuffled renditions for each cell. *N* values for all parts of this figure are stated in Table 1.

An analysis of the proportion of cells participating in sequences is presented in Fig. 3, with statistical details provided in Supplemental Table S1. The left panel of Fig. 3*A* shows a comparison of the proportions of spikes in sequences in cells recorded in the normal and parkinsonian states. The tendency to fire in sequences was almost twice as high in the pallidum than it was in the STN in the normal state. After induction of parkinsonism, this proportion dropped significantly in the GPe cells, whereas it increased significantly in neurons recorded in the STN. There was no change in this parameter in the GPi recordings. In the right panel of Fig. 3*A*, we compared the proportions of spikes found in sequences with the median of the proportion of spikes in sequences based on shuffled representations of each cell. In all cases, we found that the proportion of sequences in the recorded arrangement of ISIs was significantly more common than in their shuffled representation suggesting that the cellular activity favored the occurrence of sequences.

**Figure 4.**
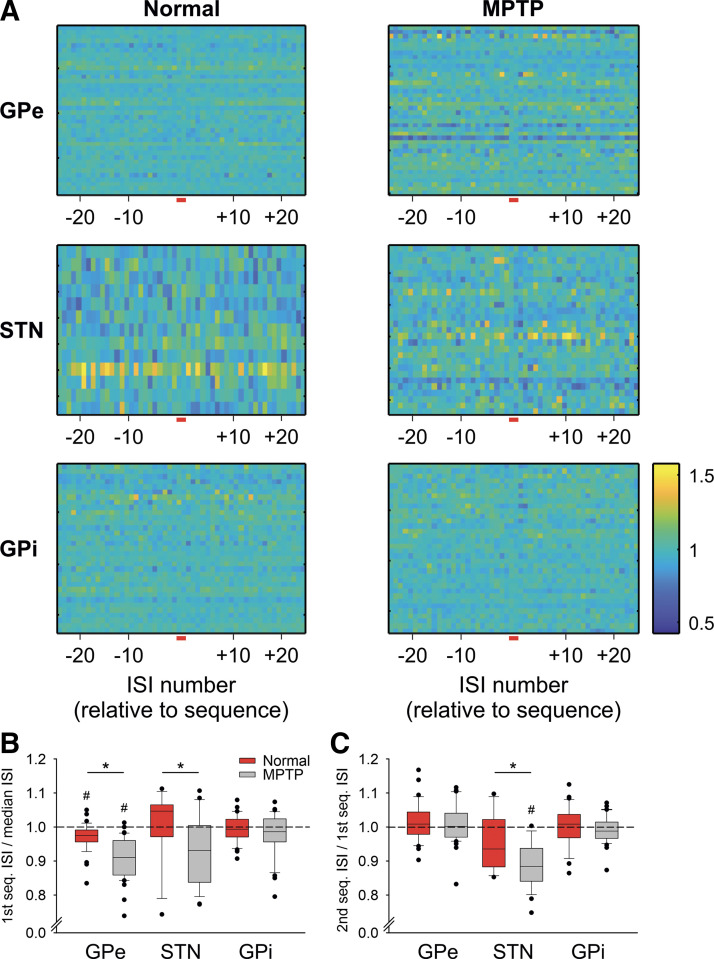
Comparison of the duration of sequence interspike intervals (ISIs) with the cell’s median ISI duration. The data are based on an analysis of 2-ISI sequences within the first 5,000 ISIs in each data file. The plots in *A* were created by normalizing a given cell’s ISIs to its median ISI. We then extracted data segments starting 25 ISIs before the start of sequences and lasting until the 25th ISI after each sequence and calculated the median across all sequences. Each line in the color plots corresponds to these values from a single neuron. The short red lines underneath the abscissas indicate the location of sequences. The plots on the *left* show data from external pallidal segment (GPe), subthalamic nucleus (STN), and internal pallidal segment (GPi) from the normal state, and those on the *right* show data from the parkinsonian state. *B*: the median of all 1st ISIs of the normalized 2-ISI sequences shown in *A* from the normal (red) and parkinsonian states (gray). *C*: the median ratios between the second and first ISI of the sequences. In *B* and *C*, the boxes show the 25th and 75th percentile ranges, with the median marked inside of the box, the error bars show the 10th and 90th percentile, and the black dots indicating values outside of the 10th/90th percentile boundaries. *P < 0.05; Mann–Whitney tests were used to compare the data from the normal and parkinsonian states. #*P* < 0.05; Wilcoxon tests were used for comparisons of the original data with the corresponding shuffled data representations. *N* values for all parts of this figure are stated in Table 1.

An analysis of the time spent in sequences (shown in Fig. 3*B*) revealed results similar to those found in Fig. 3*A*. In Fig. 3*C*, the duration of sequences is compared. As this measure would obviously differ between sequences containing different numbers of spikes, we restricted this analysis to the most common type, i.e., sequences containing three spikes. This analysis showed that sequences had similar lengths in the GPe and GPi samples from the normal and parkinsonian states but were longer in duration in the STN in the normal state than in the parkinsonian state. Comparison with shuffled data showed that the sequences were shorter in the recorded data in GPe and GPi in both, the normal and parkinsonian states, and in the STN data in the MPTP-treated state. This suggests that one or both ISIs in the sequence data was shorter than the median ISI length. This aspect of sequences was also studied in Fig. 4.

The analyses in Fig. 3 were also carried out using other sequence detection thresholds (see Supplemental Figs. S1, S2, S3, S4, and S5). As expected, the proportion of spikes in sequences and other parameters differed, depending on the detection method, but the principal findings remained the same as outlined here.

Following up on the results in Fig. 3*C*, we studied whether the ISIs within 2-ISIs sequence are episodes of brief accelerations or decelerations of firing. Using the ISIs found in the first occurrence of each such sequence, we calculated ratios between the in-sequence ISIs and the respective cell’s median ISI and then generated the median of all such ratios found within the discharge of individual cells. The results of this analysis are shown in Fig. 4*A*. The figure demonstrates that there is generally little difference between the sequence ISIs and nonsequence ISIs before and after the sequence in question. As shown in Fig. 4*B*, however, the first ISIs of detected sequences tended to be minimally (but significantly) shorter than the cell’s median ISI in the GPe and STN recordings. This finding was more pronounced in the data from the parkinsonian state in both structures. The analysis in Fig. 4*C* shows that in the STN recordings, the second ISI in the detected sequence tended to be shorter than the first. This was not the case in either of the pallidal segments.

### Relationship between the Proportion of Spikes in Sequences, the Firing Rate, and Bursts

**Figure 5.**
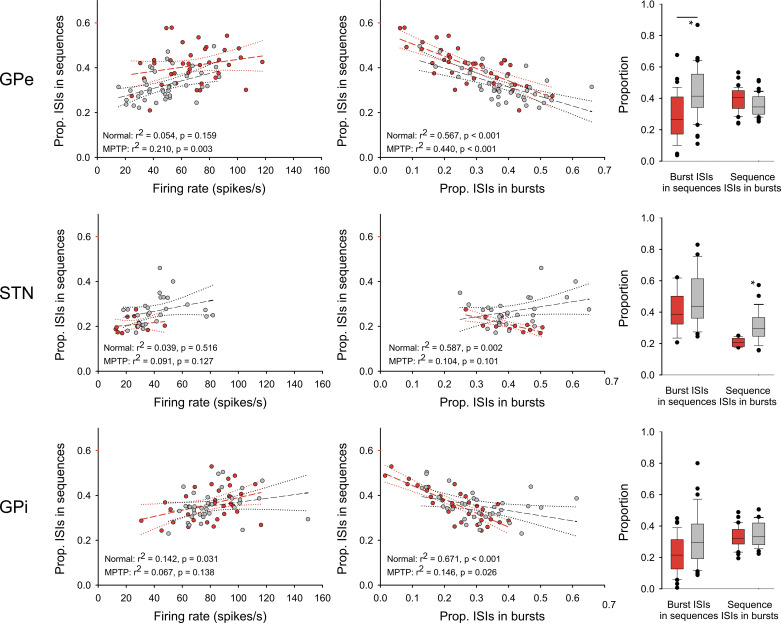
Relation between sequence and bursts. The scatter plots in the left column show the relationship between the proportion of spikes in sequences (ordinate) and the cell’s firing rate (abscissa). The middle column of plots shows the relationship between the proportion of interspike intervals (ISIs) in sequences (ordinate) and the proportion of ISIs within bursts (abcissa). Each symbol represents a single recorded neuron. The dashed lines represent the result of a linear regression analysis (*r*^2^ values and *P* values are indicated in the figures). The red symbols represent data from the normal state, while the gray symbols represent data from the parkinsonian state. The box plots on the right show the median of the overlap between ISIs that were found to be within sequences and those that were found within bursts. Error bars indicate the 25th–75th percentile ranges. The box plots on the left of these figures show the proportion of ISIs within sequences that were also found to be part of a burst. The box plots on the right show the proportion of ISIs within bursts that were also found to be part of sequences. *P < 0.05; Mann–Whitney test, comparing the data from the normal and parkinsonian states. *N* values for all parts of this figure are stated in Table 1.

We explored the relationship between sequences and the frequency of firing and the occurrence of bursts with the analysis shown in Fig. 5. The proportion of spikes in sequences was either weakly or not significantly correlated with the firing rate (left column of plots). For almost all groups, there was a significant correlation to bursts (middle column of plots). In most of the groups, the proportion of spikes in bursts and those found in sequences were inversely correlated (i.e., more sequences were found in cells that showed less bursting). The exception to this were findings in the STN after treatment with MPTP, where high numbers of bursts correlated with higher numbers of sequences. However, this correlation was not significant.

The relationship between bursts and sequences was further explored with the analysis shown in the right column of Fig. 5. The boxplots show the median and interquartile range of the proportion of burst ISIs among ISIs that were found in sequences (left box plots) and the proportion of sequence ISIs among those ISIs that were found to be in bursts. Twenty to forty percent of ISIs were found in both populations of ISIs. The proportion of burst ISIs among sequence ISIs was increased in the GPe sample in the parkinsonian state, as was the proportion of sequence ISIs among burst ISIs in the STN sample. There were no other significant changes between the normal and parkinsonian states.

## DISCUSSION

The presented results demonstrate that repeating sequences of ISIs are more common than expected by chance in the spontaneous firing of basal ganglia neurons in GPe, GPi, and STN, in the healthy and the parkinsonian states. Two-ISI sequences were the most numerous. These sequences tended to occur in clusters, and ISIs within ISIs were, on average, shorter than the median ISI of the cell. These findings suggest that the formation of such sequences is a feature of neuronal discharge in the basal ganglia. We also found that the proportion of spikes in sequences changes in GPe and STN in the dopamine-depleted, parkinsonian state, with a reduced probability of finding sequences in GPe and an increased probability of finding them in the STN. The MPTP treatment did not alter the proportion of spikes in sequences of data collected from GPi. We also show that although there is overlap between the group of ISIs in bursts and those in sequences, these phenomena are not strongly related to one another.

### Parameters That Determine Sequence Detection

Several factors determine the results of sequence detection by the methods used in our study. The length of the recordings is obviously important, with longer recordings favoring the detection of sequences, likely because sequences that occur less frequently can be detected if more time is allowed for their (re-)occurrence. The detection threshold is another important variable in this analysis. For our main analysis, we identified a series of ISIs as being a repetition of an earlier ISI series (and thus, a sequence) if all members of the sequence were no more than 1% (0.01) different from the corresponding members of the original sequence (see Supplemental Figs. S1, S2, S3, S4, and S5 for results using other detection thresholds). For all thresholds used, the detection criteria are temporally more stringent for sequences that contain short ISIs, so that results from nuclei with greatly differing average firing rates (such as the STN and the GPi) may not be fully comparable. However, as shown in Fig. 5 (left column), there was no strong relationship between the average firing rate of neurons and the proportion of spikes in sequences in that neuron (in fact, neurons with a higher firing rate were, if anything, slightly more likely to generate sequences than cells with a low firing rate).

The choice of other parameters also substantially influenced the result. As could be expected, the length and the cut-off criterion both influenced the likelihood of finding sequences. Sequences in the original data occurred overall more frequently than predicted by chance (see Fig. 3, *right*). Furthermore, there is an obvious interaction between the detection of sequences in the original and the shuffled data streams, as ISIs that repeatedly appear in the original data stream would end up being more common in general, thus, favoring random ISI arrangements containing such sequences. Our method of testing whether the original sequence detection was explained by random arrangement of ISIs alone is therefore a conservative estimate.

We also examined how stable estimates of the likelihood of finding spike sequences would be with a given length of ISI stream. Not surprisingly, the use of short data segments resulted in variable estimates, whereas long data segments provided more stable results (Fig. 1*B*). As a compromise between these results, we used ISI data streams that were 5,000 ISIs in length.

### Comparison to Literature Findings

Precisely replicating patterns of spikes have been described in a variety of other brain locations, mostly belonging to visual or olfactory domains (19, 21–23, 30). As mentioned by other authors, replicating patterns in these systems may present temporal coding of precisely patterned inputs (31) or may represent coded symbols (21, 23). Although it cannot be excluded that such temporal or symbolic coding also occurs in the “spontaneous” state, the presence of such patterns in studies of spontaneous discharge [as in our study and others (19)] suggests that the patterning of firing is triggered, at least under some circumstances, by (unknown) intrinsic biophysical cellular or network activities.

In sensory systems, repeating ISI patterns in cortical recordings were more common than expected when measured against Monte Carlo-simulated random arrangements of spikes, as also done in our study (22, 31). We found, however, that the likelihood of finding sequences in spontaneous firing data from the basal ganglia did not reach the same level of significance as those found in the studies of cortical firing. This suggests that external stimuli are represented with less temporal precision in the basal ganglia, or that cellular/network properties of basal ganglia neurons are less conducive to sequence coding than neurons in the cerebral cortex.

In most studies, including ours, the detected sequences do not extend over long periods. By far, most of the detected patterns consisted of short repetitions (triplets or quadruplets), and, commensurate with the average firing rate of the individual nuclei, the average duration of the detected repeating series of spikes (for the most common type of sequence comprising 3 spikes) did not extend beyond 25 ms in either segment of the globus pallidus, and 50 ms in the STN (as shown in Fig. 3*C*).

As shown in Fig. 3*A*, there were substantial differences in the sequence detection rates between GPe, STN, and GPi, which grossly parallel the differences in median firing rates across these nuclei. Because we did not explicitly correct our analysis for such differences in firing rates (see, e.g., Ref. 19), the absolute sequence detection rates between nuclei have to be evaluated with caution. We note, however, that within nuclei and states, there were only weak relationships between firing rates and sequence detection (Fig. 5).

### Biological Relevance

Between 20% and 40% of all spikes in the GPe, GPi, and STN were found to be part of sequences, as defined here (using the 1% criterion). Our animals were recorded in the “spontaneous” awake state. We have no concrete information that would help us under these circumstances to assess the encoding properties of the spikes. In previous studies of the occurrence of sequence (see previous section), sequences were interpreted as encoding recurrent sensory inputs or motor patterns, but any such inference for the current data would be speculation. Although it seems likely that the tendency to discharge in sequences under rest conditions is related to a given cell’s membrane properties or channel endowment, it is not clear how sequences originate. We did not observe a strong overlap with bursts (sequences could occur within or outside of bursts), thus it is not likely that the two are explained by the same phenomena.

The finding of sequence alterations in the parkinsonian state adds another abnormality to the list of findings that separates parkinsonian from healthy animals. So far, single-cell firing in STN, GPe, and GPi was found to be characterized by changes in firing rates and the occurrence of (oscillatory and nonoscillatory) bursts (3). The presented findings suggest that, in the parkinsonian state, sequences occur less commonly in GPe and more commonly in the STN. The fact that the occurrence of sequences was accentuated in the parkinsonian state may suggest that dopamine loss [or secondary synaptic changes (e.g., see Refs. 32 and 33)] may favor biophysical changes in the networks that incorporate the basal ganglia that may then be conducive to sequence generation.

### Limitations

The most significant limitation of this study is its small sample size. We used the opportunity to analyze data that were available from a different study (see *General Study Design* under METHODS). Ideally, the analysis would have included a larger number of cells from each structure. Even with these limitations in mind, however, the current study allowed us to show that sequence formation does occur in the extrastriatal basal ganglia and that the tendency to show sequences is altered by the parkinsonian state.

Another limitation is the fact that we only recorded cells in the spontaneous state. This state was defined by the presence of wakefulness and the absence of overt movements, but this does not rule out that sensory- or motor-related information was processed. It is possible that repeating sequencing is an “idling” phenomenon that would be suppressed when movements are underway. Alternatively, sequences may be part of the information processing currency used by cells to communicate with each other. As mentioned earlier, it would be highly interesting whether sequences encode external events (e.g., become more or less common in animals performing a task).

Finally, given the prominence of changes in synchrony of firing in the basal ganglia in the parkinsonian state, it would have been interesting to simultaneously record neighboring cells and to study whether they generate similar or separate sequences. It would be expected that the similarity of sequences in neighboring cells would increase with the onset of parkinsonism. We were not able to test this hypothesis, given the nature of the available dataset.

### Summary

To our knowledge, the current report is the first documentation of repeating patterns within series of basal ganglia ISIs and is the first time that the relationship between parkinsonism (or other disease states) and repeating sequences of neuronal spikes has been explored. The generation of sequences may be an important method of information transfer between these nuclei which, when disturbed, as apparent in parkinsonian animals, may contribute to behavioral abnormalities. Future delineation of the biophysical mechanisms underlying the generation of sequences in basal ganglia discharge may help us to better define their relevance for the function of this firing behavior under normal and disease states.

## DATA AVAILABILITY

All source data are available at https://doi.org/10.5281/zenodo.6581800. The Matlab codes used to identify sequences and to analyze patterns within the ISI data are available at https://zenodo.org/badge/latestdoi/496319009.

## SUPPLEMENTAL DATA

10.5281/zenodo.7724104 Supplemental Table S1 and Supplemental Figs. S1–S5: https://doi.org/10.5281/zenodo.7724104.

## GRANTS

Funding for this analysis was provided by NIH Grants P50NS098685 and P50NS123103, and an infrastructure grant to the Emory National Primate Research Center under Grant No. P51OD011132. This research was also funded in part by Aligning Science Across Parkinson’s [ASAP-020572] through the Michael J. Fox Foundation for Parkinson’s Research (MJFF).

## DISCLOSURES

No conflicts of interest, financial or otherwise, are declared by the authors.

## AUTHOR CONTRIBUTIONS

T.W. conceived and designed research; T.W. performed experiments; A.G. and T.W. analyzed data; A.G. and T.W. interpreted results of experiments; T.W. prepared figures; T.W. drafted manuscript; A.G. and T.W. edited and revised manuscript; A.G. and T.W. approved final version of manuscript.
